# Inflammatory gene variants and the risk of biliary tract cancers and stones: a population-based study in China

**DOI:** 10.1186/1471-2407-12-468

**Published:** 2012-10-11

**Authors:** Felipe A Castro, Jill Koshiol, Ann W Hsing, Yu-Tang Gao, Asif Rashid, Lisa W Chu, Ming-Chang Shen, Bing-Shen Wang, Tian-Qua Han, Bai-He Zhang, Shelley Niwa, Kai Yu, Hong Zhang, Stephen Chanock, Gabriella Andreotti

**Affiliations:** 1 Division of Cancer Epidemiology and Genetics, National Cancer Institute, National Institutes of Health, 6120 Executive Blvd., EPS 7063, Rockville, MD, 20892, USA; 2 Shanghai Cancer Institute, Shanghai, China; 3 Department of Pathology, MD Anderson Cancer Center, Houston, TX, USA; 4 Shanghai Tumor Hospital, Shanghai, China; 5 Zhongshan Hospital, Fudan University, Shanghai, China; 6 Department of Surgery, Ruijin Hospital, Shanghai Second Medical University, Shanghai, China; 7 Institute of Oriental Hepatobiliary Surgery, Second Military Medical University, Shanghai, China; 8 Westat, Rockville, MD, USA; 9 Institute of Biostatistics, School of Life Science, Fudan University, Shanghai, China; 10 Laboratory of Translational Genomics, Division of Cancer Epidemiology and Genetics, National Cancer Institute, National Institutes of Health, Advanced Technology Center, Bethesda, Maryland, 20892-4605, USA

**Keywords:** Biliary tract cancer, Biliary stones, Inflammation, Genetic susceptibility

## Abstract

**Background:**

Genetic variants in inflammation-related genes have been associated with biliary stones and biliary tract cancers in previous studies.

**Methods:**

To follow-up on these findings, we examined 35 single nucleotide polymorphism (SNPs) in 5 genes related to inflammation (*IL8*, *NFKBIL*, *RNASEL*, *TNF*, *and VEGFA*) in 456 participants with incident biliary tract cancer cases (262 gallbladder, 141 extrahepatic bile duct, 53 ampulla of Vater), 982 participants with biliary stones, and 860 healthy controls in a population–based case–control study in Shanghai, China.

**Results:**

Suggestive associations were observed for SNPs in *VEGFA* with biliary stones, *IL8* with gallbladder and ampulla of Vater cancers, and *RNASEL* with ampulla of Vater cancer (false discovery rate≤0.2).

**Conclusion:**

These findings provide additional support for the role of inflammation in biliary stones and biliary tract cancer risk and need further validation.

## Background

Biliary tract cancers, which include cancers of the gallbladder, extrahepatic bile duct, and ampulla of Vater, are rare, yet highly fatal malignancies [1]. Elevated incidence rates have been reported in Native Americans and Hispanic immigrants in the United States, certain populations in Asia (including China, Korea, Japan and India), and in some parts of Eastern Europe and South America [2]. Previous clinical and population-based studies have linked various inflammatory factors and mechanisms with the development of biliary tract cancers [3-6]. For example, gallstones, the predominant risk factor for biliary tract cancers, are thought to cause repeated irritation of the biliary tact mucosa, leading to chronic inflammation and eventual malignant changes [2].

Variants in genes involved in inflammatory pathways have been linked to biliary tract cancer and biliary stones. For example, studies in India, a high-risk population for gallbladder cancer, have shown that polymorphisms in *IL1*[7], *TNFalpha*[8], and *CCR5*[9] are associated with gallbladder cancer. Data from our population-based study of biliary tract cancers in Shanghai suggested that variants in *PTGS2*[10], *IL8*, *IL8RB*, *RNASEL*, *NOS2* and *VEGF* were associated with biliary tract cancer and/or stones [11]. To follow-up on these initial findings, in this analysis we examined an additional 28 SNPs in four of the candidate genes we previously identified (*IL8*, *RNASEL*, *TNF*, and *VEGFA*) in our population-based study in Shanghai. We also evaluated five SNPs in *NFKBIL*, a novel gene in the major histocompatibility complex (MHC) class I region that was not evaluated previously.

## Methods

### Study participants

The study protocol was approved by the Institutional Review Boards of the USA National Cancer Institute and Shanghai Cancer Institute. All participants provided written informed consent. The methods of the Shanghai Biliary Tract Cancer Study have been reported [11]. Briefly, cases were residents of urban Shanghai between the ages of 40 and 74. They were diagnosed with biliary tract cancer (ICD-9 156) between 1997 and 2000, and identified at 42 collaborating hospitals. Biliary stone cases without a history of cancer were ascertained from the same hospitals and matched to index cancer cases on hospital, gender, and age (within 5 years). Biliary tract cancer and biliary stone cases were confirmed by an expert panel review of clinical and pathology records. Population controls were healthy subjects without a history of cancer selected from the Shanghai Resident Registry and frequency-matched to cancer cases in a 1-to-1 ratio by gender and age (5-yr intervals). Biliary stone status among population controls was assessed by abdominal ultrasound or self-reported history. Cases were interviewed within three weeks of diagnosis. The interview response rate was over 95% for cases and 82% for controls.

### Gene and SNP selection and genotyping

The variants included in this analysis were chosen based on *a priori* evidence for a role in the immune/inflammatory response [12,13] or biliary disease [11]. We selected 35 SNPs in 5 genes, including 30 SNPs in the candidate genes identified in our previous study (*IL8*, *RNASEL*, *TNF* and *VEGFA*), as well as 5 SNPs in the *NFKBIL* gene that was not evaluated previously (Table 1). Genomic DNA was extracted from buffy coat using phenol-chloroform extraction. All genotyping was performed by TaqMan (Applied Biosystems, Foster City, CA) at the National Cancer Institute Core Genotyping Facility (CGF, Advanced Technology Corporation, Gaithersburg, MD) (http://cgf.nci.nih.gov/home.cfm). Eight samples were randomly selected as quality control (QC) samples; six replicates from each sample were interspersed among genotyping assays and blinded to laboratory personnel. There was 100% concordance among the QC specimens, and the genotyping completion rate for the SNPs was 98%.

**Table 1:** Selected inflammatory genes and their association with biliary stones and cancer in the Shanghai population

			**Evaluated SNPs**		
**Gene symbol** (**name**)	**Gene function**	**Chromosome location**	**Examined in the current study**	**Previously reported variants**^**1**^	**High linkage disequilibrium**
*IL8* (*interleukin 8*)	Neutrophil chemotaxis	4q12-q13	rs12506479, rs10805066 rs2227543, rs7657356	rs4073, rs2227307, rs2227306	rs2227307, rs4073, rs2227543 and rs2227306 (r^2^>0.8)
*NFKBIL* (*nuclear factor of kappa light polypeptide gene enhancer in B*-*cells inhibitor*-*like 1*)	Encodes divergent member of I-kappa-B family proteins	6p21.3	rs2230365, rs2239707, rs2857605, rs928815, rs13215091		
*RNASEL* (*ribonuclease L*)	Encodes an interferon-inducible ribonuclease	1q25	rs11807829, rs474939, rs533259, rs682585, rs627839, rs627928, rs635261, rs672527, rs579006	rs11072, rs486907	rs11807829 and rs11072 (r^2^=0.98), rs635261 and rs627839 (r2=0.8), rs579006 with rs486907 (r2=0.95)
*TNF* (*tumor necrosis factor*)	Inflammatory cytokine that promotes hyperlipidemia by increasing hepatic triglyceride production and decreasing clearance	6p21.3	rs2857708, rs769177, rs769178	rs1800750, rs1800629, rs361525, rs673, rs1799724, rs1800630, rs1800610, rs1799964	rs769178 and rs1799724 (r^2^=0.95)
*VEGFA* (*vascular endothelial growth factor A*)	Vascular permeability, angiogenesis, vasculogenesis, cell growth, cell migration, apoptosis	6p12	rs25648, rs3025000, rs3025033, rs3025035, rs833052, rs866236, rs9367173, rs9394963, rs998584, rs10434, rs6905288, rs6899540, rs4714696, rs833070	rs3025039	(rs3025033 and rs3025039 (r^2^=0.93)

^1^Previously reported variants associated with the risk of biliary stones and/or biliary tract caner in the Shanghai population study.

### Statistical analysis

The final analysis included subjects who completed the interview and for whom we had DNA samples and genotyping results. A total of 456 biliary tract cancer cases (262 gallbladder, 141 extrahepatic bile duct and 53 ampulla of Vater), 982 biliary stone cases (252 bile duct, 730 gallstone), and 860 controls, were included. Hardy-Weinberg equilibrium of allele frequencies for all SNPs was tested among controls using the chi-square test. Unconditional logistic regression was conducted to estimate odds ratios (OR) and 95% confidence intervals (CI) for the associations between SNPs (using additive and co-dominant genetic models) and biliary stone and cancer risks. Biliary stone cases were compared with healthy controls without stones; gallbladder cancer cases were compared with population controls without a history of cholecystectomy; and bile duct and ampulla of Vater cancer cases were compared with all population controls. Statistical interactions between SNPs and biliary stones were examined using the likelihood ratio test in a logistic regression model. Risk estimates were adjusted for age (categorical) and sex, and further evaluated for other potential confounding factors. To account for multiple comparisons, we used the Benjamini-Hochberg method to control for the false discovery rate (FDR) [14], considering a FDR≤0.2 being noteworthy, as per previous studies [15,16]. We also calculated a gene-based summary p-value using the minP test [17] for each of the five genes using the SNPs examined in this analysis and the SNPs from our previous study [11].

## Results

The distribution of selected characteristics among cases and controls used in the current analysis (Additional file 1: Table S1) is similar to the distributions reported previously (11). Of the 35 SNPs examined, statistically significant (p<0.05) associations were seen for *VEGFA* rs9367173 and rs6905288 with biliary stones, *IL8* rs10805066 with gallbladder and ampulla of Vater cancers, and *RNASEL* rs672527 with ampulla of Vater cancer (Table 2). Individual SNP-associations are shown in Additional file 2: Table S2. Adjustment for biliary stones did not change the effect of these SNPs on cancer, except for *IL8* rs10805066 and gallbladder cancer, which was no longer statistically significant (data not shown). Stratifying by biliary stone status did not identify additional associations. The associations noted above were robust to multiple comparisons at the FDR p≤0.2 level, with FDR p-values ranging between 0.09 and 0.2. Including SNPs from this analysis and our previous study [11] in the minP test did not result in additional significant gene-based associations (Additional file 3: Table S3).

**Table 2:** **Odds Ratios** (**ORs**) **and 95**% **Confidence Intervals** (**CIs**) **for the association between inflammatory variants and biliary stones**, **gallbladder and ampulla of Vater cancers in the Shanghai population**

**SNPs**	**rs**#	**Controls**^**1**^**n(%)**	**Casesn(%)**	**OR**^**2**^ (**95**% **CI**)	**p**^**3**^	**FDR p**^**4**^
**Biliary stones**						
*VEGFA* 9921bp 3' of STP A>G	rs9367173					
GG		539 (82.9)	837 (86.8)	1.0		
AG		102 (15.7)	126 (13.1)	0.8 (0.60-1.07)		
AA		9 (1.4)	6 (0.6)	0.4 (0.14-1.15)		
*trend*^*3*^					***0***.***03***	***0***.***2***
AG+AA		111 (17.1)	132 (13.7)	0.77 (0.58-1.01)		
*VEGFA* 6507bp 3' of STP G>A	rs6905288					
AA		357 (55.3)	499 (51.8)	1.0		
AG		249 (38.5)	386 (40.0)	1.14 (0.92-1.41)		
GG		40 (6.2)	81 (8.4)	1.5 (1.00-2.25)		
*trend*^*3*^					***0***.***04***	***0***.***2***
AG+GG		289 (44.7)	467 (48.4)	1.19 (0.97-1.46)		
**Gallbladder cancer**						
*IL8* -13985C>G	rs10805066					
CC		647 (81.1)	187 (73.0)	1.0		
CG		140 (17.5)	68 (26.6)	1.67 (1.20-2.34)		
GG		11 (1.4)	1 (0.4)			
*trend*^*3*^					***0***.***03***	***0***.***2***
CG+GG		151 (18.9)	69 (27.0)	1.57 (1.13-2.18)		
**Ampulla of Vater Cancer**						
*RNASEL* IVS5+170G>A	rs672527					
GG		712 (83.6)	39 (73.6)	1.0		
AG		133 (15.6)	11 (20.8)	1.54 (0.77-3.10)		
AA		7 (0.8)	3 (5.7)	8.40 (2.06-34.14)		
*trend*^*3*^					***0***.***01***	***0***.***09***
AG+AA		140 (16.4)	14 (26.4)	1.88 (0.99-3.57)		
*IL8* -13985C>G	rs10805066					
CC		685 (80.4)	37 (69.8)	1.0		
CG		156 (18.3)	13 (24.5)	1.59 (0.82-3.07)		
GG		11 (1.3)	3 (5.7)	5.6 (1.47-21.42)		
*trend*^*3*^					***0***.***02***	***0***.***1***
CG+GG		167 (19.6)	16 (30.2)	1.83 (0.99-3.39)		

^1^ Gallbladder cancer cases compared with population controls who had a gallbladder, Ampulla of Vater cancer cases compared with all population controls and Biliary stone cases compared with population controls who did not have biliary stones; ^2^ Adjusted for gender and age group; ^3^ Test of trend for genotype under additive model; ^4^ Adjusted by using the Benjamini-Hochberg method.

## Discussion

Following-up on findings suggesting an association between inflammation-related genes and biliary stones and cancer [11], in this analysis we expanded the gene coverage of four previously identified genes and examined another gene not previously studied in the Shanghai Biliary Tract Cancer Study. We observed suggestive associations for SNPs in *VEGFA* with biliary stones, *RNASEL* with ampulla of Vater cancer, and *IL8* with gallbladder and ampulla of Vater cancers after correcting for multiple comparisons (FDR≤0.2).

Genetic susceptibility to biliary stones was linked to two SNPs in *VEGFA*. These two SNPs, rs9367173 and rs6905288, are located downstream of *VEGFA* and are neither in linkage disequilibrium (LD) with each other, nor in LD with the other *VEGFA* SNPs we examined. VEGFA is a signal protein that is fundamental in vascular permeability and angiogenesis [18]. Thus, the role of *VEGFA* in gallstones susceptibility could be attributed to the process of blood vascularization during acute and/or chronic inflammation; however, the functional effects of these variants are unknown. To our knowledge, this study is the first to report an association between *VEGFA* and biliary stones. In our previous study, *VEGFA* was not associated with biliary stones, but rather *VEGFA* rs3025039 was associated with gallbladder cancer. This variant is not in LD with the two *VEGFA* SNPs we identified in this analysis.

We also found an increased risk of ampulla of Vater cancer with *RNASEL* rs672527, which is located in an intronic region of the gene. RNASEL is a key component of the innate immune system and participates in the process of apoptosis [19], but the functional effect of rs672527 is unknown. Other polymorphisms in the *RNASEL* gene have been associated with an increased risk of such cancers as prostate, head and neck, uterine cervix and breast [20,21]; however, this is the first report to our knowledge of an association of rs672527 with cancer. We have previously shown that another *RNASEL* SNP (rs486907) was associated with biliary stones. These two SNPs are not in LD.

*IL8* rs10805066, which was linked with increased risks of ampulla of Vater and gallbladder cancers, is located outside the promoter region of the gene. No functional effects of the variant have been reported. IL8 chemokine has been recognized as a potent mitogenic/angiogenic and inflammatory factor [22], which could support its participation in biliary tract cancer development. In our previous study, other variants in *IL8* (rs4073, rs2227307 and rs27306, all in LD) were associated with bile duct stones [11]. These three SNPs are not in LD with rs10805066.

The associations between *RNASEL* and *IL8* variants with biliary tract cancers were independent of biliary stones. In our previous study, some, but not all genetic variants interacted significantly with biliary stone status to effect biliary cancer risk (Hsing, 2008). It has been suggested that most of the inflammatory processes of biliary tract cancer are linked to biliary stones; however, not all biliary cancer cases have prior stones, and other inflammatory conditions such as cholecystitis, history of gastric or duodenal ulcers have been reported to play a role [23]. The cascade of inflammatory events in relation to the genes detected in this or previous studies, with or without the presence of biliary stones is unclear. In general, in both humans and mouse models, inflammatory processes, such as edema of the gallbladder, increased organ wall thickness, inflammatory infiltrates (the presence of inflammatory cells), and increases in transforming growth factor (TGF)-β production [24]) lead to chronic inflammation [25,26], which may eventually lead to carcinogenesis.

Although we extended the analysis of common genetic variants to approximately 80% coverage for *VEGFA* (chr6: 43827369–43870265), 90% for *RNASEL* (chr1: 180805238–180826133) and 75% for *IL8* (chr4: 74831698–74808648), none of the overall effects for each of the tested genes resulted in significant associations at the 0.05 level with either biliary stones or cancer. Our findings from this analysis, together with our findings from our previous study conducted in the same population do not strongly support an association between a particular gene and a particular biliary disease. It may be possible that each of the current and previously examined SNPs have independent effects on stones or biliary tract susceptibility, which is supported by the absence of LD (D’ or r^2^) between the SNPs. In addition, these variants may influence expression of different genes, which it is supported by the findings of recent follow-up of genome-wide association studies and the rare candidate genes [27,28]; however, we did not find evidence of regulatory consequences for the associated variants.

Strengths of this study include the population-based design, the high case ascertainment and response rates, and the detailed review of pathology and clinical data to confirm the diagnosis of cancer cases. The use of ultrasound among controls also minimized misclassification of gallstones. Although, this study is the largest population-based study of biliary tract cancer to date, we have limited statistical power to detect a modest association, in particular for cancer of the ampulla of Vater.

## Conclusion

In conclusion, after evaluating a new set of SNPs in *a priori* selected inflammatory genes, our results provide additional data to support that genetic susceptibility related to inflammatory mechanisms play a role in biliary tract cancer and stones. Further validation of these findings in other populations with full coverage of the genetic variability of each of the genes would help clarify the role of these genes in biliary tract carcinogenesis.

## Competing interests

The author declare that they have no competing interests.

## Authors’ contributions

Design, coordination, and execution of the study (A.W.H, G.A. and L.W.C.). Design and conduct of the Population-based case–control study (A.W.H., Y.T.G., A.R., M.C.S., B.S.W., T.Q.H., and B.H.Z.). Contractor (S.N.). Genotyping (S.C.). Data analyses (F.A.C., G.A., H.Z. and K.Y.). First draft of manuscript (F.A.C., G.A., J.K. and A.W.H.). All authors read and approved the final manuscript.

## Pre-publication history

The pre-publication history for this paper can be accessed here:

http://www.biomedcentral.com/1471-2407/12/468/prepub

## Supplementary Material

Additional file 1 **Table S1.** Selected characteristics of biliary tract cancer cases, stone cases, and controls in the Shanghai population. Click here for file

Additional file 2 **Table S2.** Associations^a^ between SNPs and biliary stones and biliary tract cancers based on the additive model in the Shanghai population. Click here for file

Additional file 3 **Table S3.** Selected inflammatory genes and their association with biliary stones and cancer in the Shanghai population. Click here for file
