# Supplementary material for: Inflammatory gene variants and the risk of biliary tract cancers and stones: a population-based study in China
Source: BMC Cancer. 2012 Oct 11;12:468. doi: 10.1186/1471-2407-12-468 (PMC3524039; doi:10.1186/1471-2407-12-468)
Supplement: Additional file 1 — Table S1. Selected characteristics of biliary tract cancer cases, stone cases, and controls in the Shanghai population. [file 1471-2407-12-468-S1.doc]

**Supplementary table 1. Selected characteristics of biliary tract cancer cases, stone cases, and controls in the Shanghai population**

|  |  |  |  |  |  |  |  | **Biliary Tract Cancer (n=456)** | | | | | | **Biliary Stones (n=982)** | | | |
| --- | --- | --- | --- | --- | --- | --- | --- | --- | --- | --- | --- | --- | --- | --- | --- | --- | --- |
|  |  |  |  |  |  |  |  | **Gallbladder** | | **Extrahepatic** | | **Ampulla of** | | **Gallbladder** | | **Bile Duct** | |
|  |  | **Controls^a^** | | **Controls^b^** | | **Controls^c^** | | **Cancer** | | **Bile Duct** | | **Vater** | | **Stones** | | **Stones** | |
| **Characteristics** | | **n** | **%** | **n** | **%** | **n** | **%** | **n** | **%** | **n** | **%** | **n** | **%** | **n** | **%** | **n** | **%** |
| **Total** | | 806 | 100 | 860 | 100 | 655 | 100 | 262 | 100 | 141 | 100 | 53 | 100 | 730 | 100 | 252 | 100 |
| **Gender** | |  |  |  |  |  |  |  |  |  |  |  |  |  |  |  |  |
|  | Male | 320 | 39.7 | 335 | 38.9 | 279 | 42.6 | 74 | 28.2 | 82 | 58.2 | 28 | 52.8 | 250 | 34.2 | 120 | 47.6 |
|  | Female | 486 | 60.3 | 525 | 61.0 | 376 | 57.4 | 188 | 71.8 | 59 | 41.8 | 25 | 47.2 | 480 | 65.7 | 132 | 52.4 |
| **Age** | |  |  |  |  |  |  |  |  |  |  |  |  |  |  |  |  |
|  | <55 | 112 | 13.9 | 114 | 13.3 | 102 | 15.6 | 39 | 14.9 | 22 | 15.6 | 4 | 7.5 | 231 | 31.6 | 56 | 22.2 |
|  | 55–64 | 238 | 29.5 | 248 | 28.8 | 189 | 28.8 | 64 | 24.4 | 36 | 25.5 | 13 | 24.5 | 207 | 28.3 | 77 | 30.6 |
|  | >=65 | 456 | 56.6 | 498 | 57.9 | 364 | 55.6 | 159 | 60.7 | 83 | 58.9 | 36 | 67.9 | 292 | 40.0 | 119 | 47.2 |
| **Education** | |  |  |  |  |  |  |  |  |  |  |  |  |  |  |  |  |
|  | None/Primary | 320 | 39.7 | 351 | 40.8 | 251 | 38.3 | 138 | 52.7 | 58 | 41.1 | 24 | 45.3 | 210 | 28.8 | 92 | 36.5 |
|  | Jr. and Sr. Middle | 367 | 45.5 | 382 | 44.4 | 302 | 46.1 | 99 | 37.8 | 63 | 44.7 | 24 | 45.3 | 390 | 53.4 | 119 | 47.2 |
|  | Some college | 119 | 14.8 | 127 | 14.8 | 102 | 15.6 | 25 | 9.5 | 20 | 14.2 | 5 | 9.4 | 130 | 17.8 | 41 | 16.3 |
| **Body mass index** | |  |  |  |  |  |  |  |  |  |  |  |  |  |  |  |  |
|  | <18.5 | 71 | 8.8 | 72 | 8.4 | 61 | 9.3 | 12 | 4.6 | 6 | 4.3 | 1 | 1.9 | 27 | 3.7 | 15 | 5.9 |
|  | 18.5–22.9 | 347 | 43.1 | 360 | 41.9 | 300 | 45.9 | 91 | 34.9 | 63 | 44.7 | 23 | 43.4 | 244 | 33.5 | 85 | 33.7 |
|  | 23.0–24.9 | 163 | 20.2 | 175 | 20.4 | 130 | 19.9 | 54 | 20.7 | 41 | 29.1 | 12 | 22.6 | 185 | 25.4 | 60 | 23.9 |
|  | >=25.0 | 224 | 27.8 | 252 | 29.3 | 163 | 24.9 | 104 | 39.8 | 31 | 21.9 | 17 | 32.1 | 273 | 37.4 | 91 | 36.2 |
| **Smoking** | |  |  |  |  |  |  |  |  |  |  |  |  |  |  |  |  |
|  | no | 563 | 69.8 | 602 | 70.0 | 450 | 68.7 | 191 | 73.2 | 80 | 56.7 | 30 | 56.6 | 552 | 75.6 | 160 | 63.5 |
|  | yes | 243 | 30.1 | 258 | 30.0 | 205 | 31.3 | 70 | 26.8 | 61 | 43.3 | 23 | 43.4 | 178 | 24.4 | 92 | 36.5 |
| **Drinking** | |  |  |  |  |  |  |  |  |  |  |  |  |  |  |  |  |
|  | no | 640 | 79.4 | 683 | 79.4 | 507 | 77.4 | 220 | 83.9 | 96 | 68.1 | 39 | 73.6 | 620 | 84.9 | 203 | 80.9 |
|  | yes | 166 | 20.6 | 177 | 20.6 | 148 | 22.6 | 42 | 16.1 | 45 | 31.9 | 14 | 26.4 | 110 | 15.1 | 48 | 19.1 |
| **Diabetes** | |  |  |  |  |  |  |  |  |  |  |  |  |  |  |  |  |
|  | no | 744 | 92.3 | 789 | 91.7 | 612 | 93.4 | 228 | 87.4 | 129 | 91.5 | 50 | 94.3 | 651 | 89.2 | 221 | 88.0 |
|  | yes | 62 | 7.7 | 71 | 8.3 | 43 | 6.6 | 33 | 12.6 | 12 | 8.5 | 3 | 5.7 | 79 | 10.8 | 30 | 11.9 |
| **Hypertension** | |  |  |  |  |  |  |  |  |  |  |  |  |  |  |  |  |
|  | no | 474 | 58.8 | 501 | 58.3 | 398 | 60.7 | 172 | 65.6 | 99 | 70.2 | 40 | 75.5 | 479 | 65.6 | 181 | 71.8 |
|  | yes | 332 | 41.2 | 359 | 41.7 | 257 | 39.2 | 90 | 34.3 | 42 | 29.8 | 13 | 24.5 | 251 | 34.4 | 71 | 28.2 |

^a^ Population controls without a history of cholecystectomy compare to gallbladder cancer cases; ^b^ All population controls compared to bile duct and ampulla of Vater cancer cases; ^c^ Population controls without stones compared to biliary stone cases.
