# Supplementary material for: Inflammatory gene variants and the risk of biliary tract cancers and stones: a population-based study in China
Source: BMC Cancer. 2012 Oct 11;12:468. doi: 10.1186/1471-2407-12-468 (PMC3524039; doi:10.1186/1471-2407-12-468)
Supplement: Additional file 2 — Table S2. Associationsa between SNPs and biliary stones and biliary tract cancers based on the additive model in the Shanghai population. [file 1471-2407-12-468-S2.doc]

**Supplementary table 2. Associations^a^ between SNPs and biliary stones and biliary tract cancers based on the additive model in the Shanghai population**

|  |  |  | **Genotypes** | **Biliary stones** | |  | **GBC** | |  | **BDC** | |  | **AVC** | |
| --- | --- | --- | --- | --- | --- | --- | --- | --- | --- | --- | --- | --- | --- | --- |
| **Gene** | **rs#** | **Genotypes** | **frequencies^b^** | **P-trend** | **FDR** |  | **P-trend** | **FDR** |  | **P-trend** | **FDR** |  | **P-trend** | **FDR** |
| IL8 | rs12506479 | CC/CT/TT | 522/1137/610 | 0.7 | 0.8 |  | 0.6 | 1.0 |  | **0.07** | 0.9 |  | 0.3 | 0.7 |
| IL8 | rs10805066 | CC/CG/GG | 1792/454/27 | 0.2 | 0.7 |  | **0.03** | 0.7 |  | 0.3 | 0.9 |  | **0.02** | 0.4 |
| IL8 | rs2227543 | CC/CT/TT | 805/1121/336 | 0.7 | 0.8 |  | 0.4 | 1.0 |  | 0.8 | 1.0 |  | 0.8 | 0.9 |
| IL8 | rs7657356 | CC/CT/TT | 806/1086/375 | 0.5 | 0.7 |  | 0.3 | 0.9 |  | 0.9 | 1.0 |  | 0.1 | 0.6 |
| NFKBIL | rs2230365 | CC/CT/TT | 1526/671/61 | 0.3 | 0.7 |  | 0.9 | 1.0 |  | 0.2 | 0.9 |  | 0.3 | 0.7 |
| NFKBIL | rs2239707 | CC/CT/TT | 115/849/1302 | 0.1 | 0.5 |  | 0.9 | 1.0 |  | 0.6 | 0.9 |  | 0.2 | 0.6 |
| NFKBIL | rs2857605 | CC/CT/TT | 30/476/1759 | 0.8 | 0.8 |  | 0.8 | 1.0 |  | 0.5 | 0.9 |  | 0.4 | 0.7 |
| NFKBIL | rs928815 | GG/GT/TT | 1040/981/243 | 0.5 | 0.8 |  | 0.6 | 1.0 |  | 0.5 | 0.9 |  | 0.4 | 0.7 |
| NFKBIL | rs13215091 | AA/AG/GG | 11/246/2015 | 0.3 | 0.7 |  | 0.9 | 1.0 |  | 0.9 | 1.0 |  | 0.8 | 0.9 |
| RNASEL | rs11807829 | AA/AG/GG | 1496/678/100 | 0.1 | 0.5 |  | 0.9 | 1.0 |  | 0.5 | 0.9 |  | 0.4 | 0.8 |
| RNASEL | rs474939 | CC/CT/TT | 600/1061/598 | 0.8 | 0.8 |  | 0.7 | 1.0 |  | 0.7 | 0.9 |  | 0.3 | 0.7 |
| RNASEL | rs533259 | CC/CT/TT | 2054/213/2 | 0.2 | 0.7 |  | 0.6 | 1.0 |  | 0.7 | 0.9 |  | 0.6 | 0.9 |
| RNASEL | rs579006 | CC/CT/TT | 131/819/1320 | 0.4 | 0.7 |  | 0.9 | 1.0 |  | 0.6 | 0.9 |  | 0.3 | 0.7 |
| RNASEL | rs627839 | AA/AC/CC | 215/900/1154 | 0.2 | 0.7 |  | 0.4 | 1.0 |  | 0.7 | 0.9 |  | **0.07** | 0.5 |
| RNASEL | rs627928 | GG/GT/TT | 1114/960/181 | 0.4 | 0.7 |  | 0.8 | 1.0 |  | 0.6 | 0.9 |  | 0.6 | 0.9 |
| RNASEL | rs635261 | CC/CG/GG | 283/989/995 | 0.1 | 0.6 |  | 0.2 | 0.9 |  | 0.8 | 1.0 |  | **0.09** | 0.5 |
| RNASEL | rs672527 | AA/AG/GG | 25/371/1879 | **0.07** | 0.5 |  | 0.4 | 1.0 |  | 0.2 | 0.9 |  | **0.01** | 0.4 |
| RNASEL | rs682585 | AA/AG/GG | 118/843/1312 | 0.7 | 0.8 |  | 0.5 | 1.0 |  | 0.3 | 0.9 |  | 1.0 | 1.0 |
| TNF | rs2857708 | CC/CT/TT | 1755/483/26 | **0.05** | 0.4 |  | 0.8 | 1.0 |  | 0.1 | 0.9 |  | 0.3 | 0.7 |
| TNF | rs769177 | CC/CT | 2063/196 | 0.4 | 0.7 |  | 1.0 | 1.0 |  | 0.9 | 1.0 |  | 0.7 | 0.9 |
| TNF | rs769178 | GG/GT/TT | 1650/516/42 | 0.7 | 0.8 |  | 0.7 | 1.0 |  | 0.3 | 0.9 |  | 0.9 | 0.9 |
| VEGF | rs25648 | CC/CT/TT | 1889/358/16 | 0.8 | 0.8 |  | 0.8 | 1.0 |  | 0.5 | 0.9 |  | **0.07** | 0.5 |
| VEGF | rs3025000 | CC/CT/TT | 754/1139/378 | 0.3 | 0.7 |  | 0.5 | 1.0 |  | 0.4 | 0.9 |  | 0.1 | 0.6 |
| VEGF | rs3025033 | AA/AG/GG | 1487/692/82 | 0.4 | 0.7 |  | 0.3 | 0.9 |  | 0.9 | 1.0 |  | 0.8 | 0.9 |
| VEGF | rs3025035 | CC/CT/TT | 1618/574/63 | 0.2 | 0.7 |  | 0.1 | 0.9 |  | 0.9 | 1.0 |  | **0.05** | 0.5 |
| VEGF | rs833052 | AA/AC/CC | 197/989/1082 | 0.7 | 0.8 |  | 0.7 | 1.0 |  | 0.2 | 0.9 |  | 0.9 | 0.9 |
| VEGF | rs866236 | CC/CT/TT | 547/1139/586 | 0.2 | 0.7 |  | 0.5 | 1.0 |  | 0.3 | 0.9 |  | 0.5 | 0.9 |
| VEGF | rs9367173 | AA/AG/GG | 19/310/1941 | **0.03** | 0.4 |  | **0.07** | 0.9 |  | 0.3 | 0.9 |  | **0.1** | 0.5 |
| VEGF | rs9394963 | GG/GT/TT | 1364/782/116 | 0.6 | 0.8 |  | 0.4 | 1.0 |  | **0.05** | 0.9 |  | 0.7 | 0.9 |
| VEGF | rs998584 | AA/AC/CC | 690/1133/456 | 0.7 | 0.8 |  | 0.9 | 1.0 |  | 0.6 | 0.9 |  | 0.8 | 0.9 |
| VEGF | rs10434 | AA/AG/GG | 103/760/1404 | 0.8 | 0.8 |  | 0.7 | 1.0 |  | 0.5 | 0.9 |  | **0.06** | 0.5 |
| VEGF | rs6905288 | AA/AG/GG | 1201/885/172 | **0.04** | 0.4 |  | 0.4 | 1.0 |  | 0.7 | 0.9 |  | 0.2 | 0.6 |
| VEGF | rs6899540 | AA/AC/CC | 1994/260/5 | 0.5 | 0.7 |  | 0.2 | 0.9 |  | 0.4 | 0.9 |  | 1.0 | 1.0 |
| VEGF | rs4714696 | CC/CT/TT | 128/848/1291 | 0.3 | 0.7 |  | 0.1 | 0.9 |  | 0.3 | 0.9 |  | 0.8 | 0.9 |
| VEGF | rs833070 | CC/CT/TT | 1343/802/126 | 0.4 | 0.7 |  | 0.6 | 1.0 |  | 0.6 | 0.9 |  | 0.6 | 0.9 |

GBC, gallbladder cancer; BDC, bile duct cancer; AVC, cancer of ampulla of Vater;  ^a^ Test of trend for genotype under additive genotypic model trend ; ^b^ Frequency of genotypes among both cases and controls; FDR, p-value adjusted for the Benjamini-Hochberg method.
