# Supplementary material for: Inflammatory gene variants and the risk of biliary tract cancers and stones: a population-based study in China
Source: BMC Cancer. 2012 Oct 11;12:468. doi: 10.1186/1471-2407-12-468 (PMC3524039; doi:10.1186/1471-2407-12-468)
Supplement: Additional file 3 — Table S3. Selected inflammatory genes and their association with biliary stones and cancer in the Shanghai population. [file 1471-2407-12-468-S3.doc]

Supplementary table 3. Selected inflammatory genes and their association with biliary stones and cancer in the Shanghai population

|  |  | **Min Gene p^1^** | | | |
| --- | --- | --- | --- | --- | --- |
| **Gene Symbol** |  | **Biliary Stones** | **Gallbladder Cancer** | **Bile Duct Cancer** | **Ampulla of Vater Cancer** |
|  |
| IL8 |  | 0.6 | 0.1 | 0.1 | 0.1 |
| NFKBIL |  | 0.2 | 1.0 | 0.3 | 0.3 |
| RNASEL |  | 0.3 | 0.9 | 0.9 | 0.3 |
| TNF |  | 0.2 | 0.2 | 0.8 | 0.4 |
| VEGFA |  | 0.1 | 0.4 | 0.3 | 0.1 |

^1^ Minimum-p value permutation test corrects for multiple testing accounting for correlations between SNPs within a gene (1000 permutations).
